# Systematic Approach to Negative Fukui Functions: Its Association with Nitro Groups in Aromatic Systems

**DOI:** 10.3390/ijms26010319

**Published:** 2025-01-01

**Authors:** Pedro Pablo Zamora Yates, Klaus Bieger

**Affiliations:** Departamento de Química y Biología, Facultad de Ciencias Naturales, Universidad de Atacama, Av. Copayapu 485, Copiapó 1530000, Chile; klaus.bieger@uda.cl

**Keywords:** negative Fukui function, Density Functional Theory (DFT), chemical reactivity, nitro groups

## Abstract

Fukui functions are related to electron densities, and their interpretation permits the determination of reactivity of atomic centres. However, negative values cannot be interpreted by an “electron density based” model and represent a phenomenon that has been little investigated and understood. Previous works in the literature suggest that they are related to nodes in the wave function. In our investigations we can relate negative values to nitro groups and their position on aromatic systems, as it is a moiety with HOMO electron densities close to “0” related to “electron attracting” groups. This work can help us understand and predict this phenomenon and the associated chemical reactivities. We also pay attention to the influence of the nitro group angle vs. the aromatic ring.

## 1. Introduction

The chemical reactivity of a molecule can be determined from its electronic structure, and more specifically, from the electron densities of its frontier orbitals: the highest occupied molecular orbital (HOMO) and the lowest unoccupied molecular orbital (LUMO). Analysis of the properties of these orbitals provides a pathway to understanding how molecules interact with other chemical species, offering valuable insights into which atoms or regions of a molecule are more likely to engage in chemical reactions.

A key tool for this analysis is the Fukui function that determines the local reactivity within a molecule by identifying specific atoms that are more likely to donate or accept electron pairs using electron densities of the HOMO and LUMO orbitals. Electrophilic Fukui function, $f^{-\left(\overset{⃑}{r}\right)}$, can be approximated by the density of the HOMO, $f^{-\left(\overset{⃑}{r}\right)}\;\approx \rho_{HOMO\;(\overset{⃑}{r})}$, as these high energy electrons are those that will interact with the LUMO of another moiety in an electrophilic attack. In consequence, low or negative values of $f^{-\left(\overset{⃑}{r}\right)}$ indicate a lack of reactivity [1,2,3]. However, from a physical standpoint, the electron density of the HOMO cannot be negative [2].

Thus, negative Fukui functions cannot be understood by the “above-mentioned” approximation rule as electronic densities must always be positive since they represent the probability of finding electrons at a specific point in space [1]. Nevertheless, it has been observed that in some cases $f^{-\left(\overset{⃑}{r}\right)}$ can adopt negative values that hold significance and provide important information about molecular parameters from a chemical perspective.

The current literature on the significance and implications of negative $f^{-\left(\overset{⃑}{r}\right)}\;$is limited, and its interpretation has not been thoroughly explored. Few researchers have dedicated efforts to understand its meaning and related its origin solely from a formal physical standpoint, without delving into the chemical factors that may produce negative values of this function [2], nor which atoms may show a clear tendency to adopt negative values. Therefore, there is a need for further research to elucidate this phenomenon.

From previous work we knew that nitro groups often led to low values of the Fukui function [3] in several parts of the molecule. Thus, here we aimed to investigate nitrobenzenes as model substances to generate and induce negative values of $f^{-\left(\overset{⃑}{r}\right)}\;$ to understand its origin and its relationships with frontier orbitals, its electronic implications and its connection with chemical factors. Nitro groups are strong electron attractors that strongly affect the distribution of the HOMO orbital in conjugated systems. Thus, we present a systematic approach based on nitrobenzene derivatives that helps to understand and to predict this curious phenomenon in related substances.

### Theoretical Background

Computational math as mentioned, according to Density Functional Theory (DFT), Fukui values are associated with chemical reactivity, and a moiety with high electron density in the HOMO will act as electrophile while it can be attacked by electrophiles in regions with low electron density in the LUMO [2,3].

The most common expression of the related Fukui function is [4,5]:

(1) $$f\left(\overset{⃑}{r}\right)={\left(\frac{\partial \rho \left(\overset{⃑}{r}\right)}{\partial N_{e}}\right)}_{v\left(r\right)}$$

This function presents the change in electron density when an electron is withdrawn from or added to a molecule with given geometry and constant potential. Its unit is that of electron density: electrons per volume.

For electron withdrawal and addition, Fukui function adopts two different forms when a finite difference approach is used [6,7,8]:

(2) $$f^{+\left(\overset{⃑}{r}\right)}={\left(\frac{\partial \rho \left(\overset{⃑}{r}\right)}{\partial N_{e}}\right)}_{v\left(r\right)}^{+}=\rho_{N+1}\left(\overset{⃑}{r}\right)-\rho_{N}\left(\overset{⃑}{r}\right)$$

(3) $$f^{-\left(\overset{⃑}{r}\right)}={\left(\frac{\partial \rho \left(\overset{⃑}{r}\right)}{\partial N_{e}}\right)}_{v\left(r\right)}^{-}=\rho_{N}\left(\overset{⃑}{r}\right)-\rho_{N-1}\left(\overset{⃑}{r}\right)$$

A further development based on Fukui functions is the Dual Descriptor (DD) $f^{\left(2\right)}\left(\overset{⃑}{r}\right)$ introduced by Morell et al. [9]. Mathematically, it is a derivative of the Fukui function with respect to the number of electrons:

(4) $$f^{2}\left(\overset{⃑}{r}\right)={\left(\frac{\partial f\left(\overset{⃑}{r}\right)}{\partial N_{e}}\right)}_{v\left(r\right)}={\left(\frac{\partial \rho \left(\overset{⃑}{r}\right)}{\partial N_{e}^{2}}\right)}_{v\left(r\right)}$$

This helps localize electrophilic and nucleophilic centres on a molecule. Sites with $f^{2}\left(\overset{⃑}{r}\right)>0$ will likely suffer from an electrophilic attack, while regions with $f^{2}\left(\overset{⃑}{r}\right)<0$ can be attacked by nucleophiles [9]. As the DD is less affected by orbital contraction or orbital expansion than the proper Fukui function, it is considered an even better reactivity indicator [10,11].

With a finite difference approach $f^{2}\left(\overset{⃑}{r}\right)$ can be expressed as the difference between $f^{+\left(\overset{⃑}{r}\right)}$ and $f^{-\left(\overset{⃑}{r}\right)}$ [11]:

$$f^{2}\left(\overset{⃑}{r}\right)=∆f\left(\overset{⃑}{r}\right)=f^{+\left(\overset{⃑}{r}\right)}-f^{-\left(\overset{⃑}{r}\right)}$$

(5) $$∆f\left(\overset{⃑}{r}\right)=\rho LUMO\left(\overset{⃑}{r}\right)-\rho HOMO\left(\overset{⃑}{r}\right)$$

To determine the reactivity of specific atoms in a moiety, Yang et al. introduced a condensed Fukui function as local reactivity descriptor [12,13,14]. It is defined as the change of charge on a specific atom when an electron is withdrawn from or added to the whole molecule. This can be determined via an electron population analysis according to Natural Bond Orbital (NBO) population:

(6) $$f_{k}=-{\left(\frac{{\partial q}_{k}}{\partial N_{e}}\right)}_{Vext}$$

The condensed Fukui function $f_{k}$ is normally calculated by the finite difference method with a discrete number of electrons. Thus, atomic charges are calculated for the same molecule in anionic, neutral and cationic states. Nucleophilic and electrophilic centres are established as in Equations (2) and (3):

(7) $$f_{k}^{+}=N_{k}\left(N\right)-N_{k}\left(N+1\right)$$

(8) $$f_{k}^{-}=N_{k}\left(N-1\right)-N_{k}\left(N\right)$$

Here, $N_{k}$ stands for the electronic population on atom *k* for a system with *N* + 1, *N* and *N* − 1 electrons, respectively, conserving the geometry of the neutral molecule with *N* electrons.

## 2. Results and Discussion

According to Equation (5), the Fukui function approximates the density of the HOMO, $\;f^{\left(-(\overset{⃑}{r}\right)}\;\approx \rho_{HOMO\;(\overset{⃑}{r})}$. Electronic densities should take positive values [1,3] since negative densities would not make physical sense. However, as observed in Table 1, this rule is not followed for all atoms (values marked in bold), showing that the density approximation in these cases is too coarse.

Previous investigations explain this phenomenon relating it to nodes in the HOMO [3], something that can be related to the mathematical and physical background. However, they do not relate it to functional groups nor indicate which atoms can generate a negative value for this function. For this reason, we tried to force negative values for $f^{-\left(\overset{⃑}{r}\right)}$ by chemical modifications in order to study the chemical background for this behaviour and not limit the explication to nodes. This approach can help to predict on which sites in a molecule this strange phenomenon could appear.

Since nitro groups have a high capacity to attract electrons [15], they can redistribute the HOMO in aromatic systems as well as modify the energy of the frontier orbitals, thereby reducing the band gap of both aromatic and extensively conjugated molecules [16]. We can see that for compounds **1**, **2**, **4**, **8**, **10** and **11** (Table 1) the electron-attracting effect leaves certain atoms with low HOMO density, and these are related to negative values of $f^{-\left(\overset{⃑}{r}\right)}$.

When an electron is removed from the molecule, the influence of the nucleus on the inner electrons is greater on atoms with low HOMO density, leading to contraction of these orbitals and herewith increase of the electron density. Consequently, $\rho_{N-1}\left(\overset{⃑}{r}\right)$ becomes greater than $\rho_{N}\left(\overset{⃑}{r}\right)$ (see Equation (2)), resulting in negative values for the function $f^{-\left(\overset{⃑}{r}\right)}$.

The nitrogen in the nitro group with partial positive charge is a good example of atoms with low HOMO density. In previous studies on reaction mechanisms, we found that phosphorus in oxidation state +V could be another candidate [3] but is not further investigated in this study.

Table 1 shows the results for different nitrobenzene derivatives and the effect of the position and number of the nitro groups on the HOMO and Fukui values of different atoms. Atoms that could be transformed into each other by symmetry operations are listed with the same number and differentiated by letters a, b, c etc. Symmetry elements (rotational axis or mirror planes) were included on the HOMO image as blue lines. Equivalent atoms should have the same Fukui values. Slight differences can be attributed to disorders in the molecule, e.g., by tilt in the functional group versus the molecular plane leading to conformers with lower symmetry. This leads to different interactions of the oxygen atoms with electron clouds over and under the aromatic ring as they have different phases. Even though on the first glance annoying, these derivations could help to establish a threshold for the chemical meaning of differences in Fukui values.

When not taking into account the phases of the orbitals nitrobenzene **1** has necessarily a symmetry element that passes through the nitrogen, ipso and para carbon. Thus, C2 and C2a and C3 and C2a would have forcefully the same Fukui values. When considering the phases however, nitrogroup oxygens will interact in different ways with the electrons on the upper and lower part of the ring system, and this difference will lead to different Fukui values for otherwise identical atoms.

In *o*-dinitrobenzene **2** one could expect depending on the tilt angles of the nitro groups versus the aromatic ring plane an “m” or a C_2_-symmetry with two equal nitro groups. Anyhow the most stable conformer has only one coplanar nitro moiety eliminating the symmetry and forcing all atoms to exhibit different Fukui values. The reason seems to be steric hindrance end electrostatic repulsion between oxygen atoms of the two neighboured NO_2_-groups as in 1,3-dinitrobenzene **3** the symmetry reappears and clear pairs of atoms with practically identical Fukui values can be found.

Also, the 1,4-dinitroderivative **4** shows symmetry with very similar parameters for the interchangeable atoms.

The same tendency can be found in higher nitrated derivatives: Vicinal nitro groups tend to force at least one of them to be tilted out of plane. This tilt also has influence on the Fukui values in the ring systems: Only in the coplanar conformers negative Fukui values could be observed on the ipso carbon. In some cases, it extends also the carbon in orto to the coplanar NO_2_-group.

In several cases the expected symmetry is broken due to tilt angles of the nitro groups that inhibit mirror operations. Nodal planes tend to form in a way that H-bearing C-atoms of the ring system are separated from the maximum possible amount of nitro groups. When the plane passes through an atom, the HOMO density on these atoms decreases to close to “0”, and low or negative Fukui values can be found here also and not only on the nitrogens (Based on the trend observed in this study, a HOMO density value is considered close to zero when it falls below the threshold of 0.001 eV). This leads to a strong relation of some triple substituted molecules to the electronic structure of tetra substituted analogues. Thus, electronic distribution in the 1,2,4-trinitrobenzene **6** is very similar to the 1,2,3,4-tetranitrobenzene **8** and 1,2,3-trinitrobenzene is similar to 1,2,3,5-tetranitrobenzene **9** in its electron structure. 1,3,5-trinitrobenzene **7** is a bit outstanding as it nearly preserves C_3_-symmetry on the ring system as there are three sectors observable on the aromatic ring, even though on a second glance one can observe reduction in symmetry due to a nitro group tilt. C_3_-symmetry is completely broken on the hexanitrobenzene **12** where only C_2_-symmetry can be found with the symmetry axis passing through the centre of two C-C-bonds on the ring system due to the tilt of two para-standing nitro groups.

A strange phenomenon can be observed in 1,2,3,4-tetranitrobenzene **8** where two oxygens were found without electron density in the HOMO leading to a negative Fukui value on these high electron negative atoms. Additionally, both affected nitro groups show a dihedral angle with respect to the benzene plane, so no direct mesomeric effect would be expected. Here the tendency to form a density node of the whole system through the NO_2_-groups and the ipso carbons increases the overall electron withdrawing effect of the nitro group potentiating the decrease in electron density on the atoms of these moieties, even though, strangely, the ipso-carbon is less affected then in other related structures.

The two oxygens on the central nitro group of pentanitrobenzene **11** also show negative Fukui values, and the whole group is tilted out of plane. It seems that the other moieties have stripped this group of electrons to mitigate the electron defect in the rest of the molecule and left these atoms with low HOMO electron density resulting in negative Fukui values. Thus, any reaction pass way that requires, e.g., protonation of these NO_2_-oxygen atoms will not work on this group.

Apart from these more surprising findings, the Fukui values for the different atoms correlate to those expected from the –M-effect of the functional group with negative values on carbons where a positive formal charge could be postulated.

Due to the high dependency of Fukui values on the tilt angle, we also carried out a study that correlates both parameters on nitrobenzene, comparing them also to the relative energy of the conformers.

**Figure 1:**
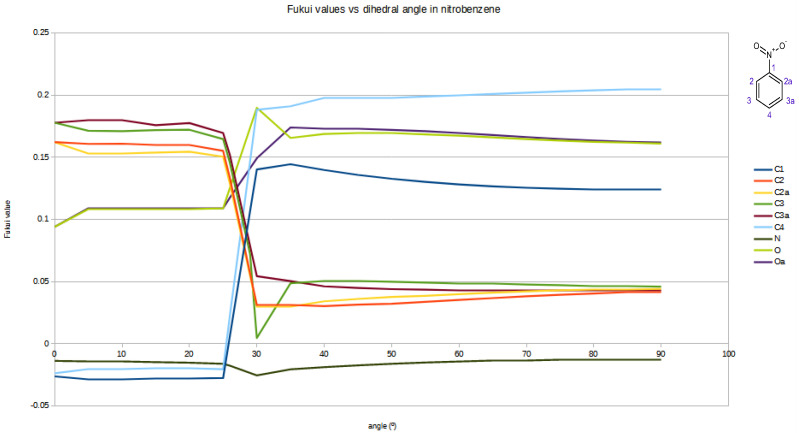
Fukui values vs. tilt angle of the nitro-group in nitrobenzene.

Astonishingly the Fukui values for nearly all atoms do not change gradually but have a harsh leap between 25 and 30° as shown in Figure 1. This change is also reflected by a change in the form of the HOMO orbital. The least affected atom is the nitrogen of the nitro group, showing clearly that the Fukui value here depends practically only on the two oxygens and is not influenced by the rest of the molecule.

Strangely the sharp change does not show up in the rotational energy where the energy profile shows a smooth graph.

The tendency of the Fukui function to approach negative values as the angle of the nitro group changes can be attributed to its role as a key descriptor of the local reactivity of the atoms within a molecule. This function reflects how the electron density responds to changes in molecular structure, particularly the distribution of electronic orbitals like the HOMO. As the angle of the nitro group is varied, the contribution of the HOMO to the electron density at specific atoms shifts, leading to a decrease in nucleophilic behaviour and an increase in the electron-attracting effect of the nucleus.

When the Fukui function takes values close to zero, it indicates that the atom’s reactivity toward electrophiles becomes negligible, as there is minimal electron density available for interaction. Similarly, negative values of the function suggest a further reduction in nucleophilic activity, potentially due to a redistribution of electron density that disfavours reactivity at that site.

As shown in Table 1 and corroborated by the Supplementary Materials Figures S1 and S2, this relationship between the angle of the nitro group and the local reactivity of the carbon atoms is consistent with how the HOMO’s distribution changes with the orientation of one or more nitro groups. Specifically, variations in the HOMO’s spatial overlap with the atom in question determine the observed trends in reactivity.

This interplay between molecular geometry and electronic structure is crucial for understanding and predicting chemical reactivity, especially in systems where substituents like nitro groups play a significant role in modulating the electron density and reactivity of nearby atoms [17,18]. Regarding the dual reactivity descriptor, as shown in Equation (5), when the Fukui function takes negative or near-zero values, the descriptor becomes more electrophilic. This occurs because, in the absence of HOMO, the atom loses its nucleophilic character and gains electrophilic character due to the presence of the LUMO.

The lower the HOMO values the lower the Fukui functions and the larger the orbital contraction. The sign-flip occurs for HOMO densities practically being zero [19]. The low HOMO density observed in the graphical representations of this orbital shows that certain atoms are completely deprived of HOMO presence, appearing “bare” or “exposed.” This leads to negative Fukui function values because the strong nuclear attraction causes the orbitals to contract, concentrating the electron density closer to the nucleus.

In cases where atoms are located at the edges or boundaries of the HOMO, the orbital’s influence is weaker. This reduced influence causes the Fukui function values to approach zero. At the edges, while some influence of the HOMO remains, the effect is minimal compared to atoms that completely lack the HOMO. Even here, the nucleus’s pull on inner orbitals still causes a slight contraction.

As the HOMO’s presence decreases—whether because an atom is at the boundary or lacks the orbital entirely—the reduced electron density lowers nucleophilic reactivity. Consequently, atoms with a deficiency of HOMO, either at the limits or in complete absence, tend to have Fukui function values that are negative or close to zero.

An aspect not yet studied to date is how the negative Fukui function $f^{-\left(\overset{⃑}{r}\right)}$ can affect the distribution of spin density. According to our calculations (Figure S3) spin density is heterogeneous, and spin redistribution is observed especially on atoms with low values or practically zero of HOMO values while the opposite spin can be localized on atoms with higher electron density in the HOMO. A possible explication is attributing it to the fact that at low electron density the probability that only one electron can be found in the region is high and one electron cannot compensate its proper spin.

## 3. Computational Methods

All calculations were carried out using the Gaussian-09 program [20]. Molecular geometries were optimized using the B3LYP-funtion on a 6-311G(d,p)—calculation base as they have proven suitable for this type of study [21,22]. The CAM-B3LYP and ωB97X-D functionals were used for compounds 6, 8, and 11 in order to determine whether dispersion effects influence the sign of the Fukui function. Figures S4 and S5 and Tables S1–S3.

For the analysis of electron population, Natural Bonding Orbitals were calculated [21], and the result was used to determine Fukui indices according to Equations (3) and (4). Gauss View 5.08, included as complement in the Gaussian 09—package, was used to generate the surfaces of the HOMO according to Equations (2) and (5).

Graphics were generated from the density with B3LYP-calculations on a 6-311G(d,p) base with isovalue 0.02.

## 4. Conclusions

We show that negative Fukui functions can not only be observed in nodes of the wave function but on any atom with HOMO values practically equal to zero helping in the understanding of this strange behaviour. The systematic approach based on nitrobenzene derivatives can even help to predict the occurrence of this phenomenon and links it to the already established rules of the −M-effect.

This work explains and helps to predict the appearance of negative Fukui functions and the related low nucleophilicity of the affected atoms. It also opens the door for further investigations that could identify other moieties with a similar behaviour.

## Figures and Tables

**Table 1:** HOMO orbital plots and Fukui values for all substances studied. The bold values indicate the negative Fukui function values.

Compound Number	Chemical Structure	HOMO	Atom Number	$f^{-\left(\overset{⃑}{r}\right)}$
**1**	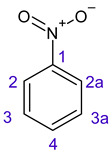	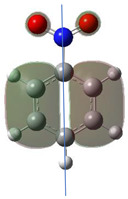	C1	**−0.02617**
			C2	0.16223
			C2a	0.16222
			C3	0.17783
			C3a	0.17783
			C4	**−0.02362**
			N	**−0.01371**
			O	0.09410
			Oa	0.09410
**2**	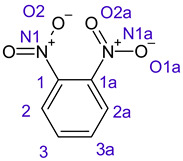	No symmetry element 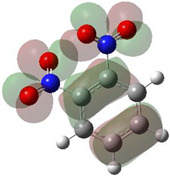	C1	0.06422
			C1a	0.06414
			C2	**−0.00185**
			C2a	0.11475
			C3	0.11459
			C3a	**−0.00275**
			N1	**−0.01226**
			N1a	0.13211
			O1	**−0.01226**
			O1a	0.13566
			O2	0.13575
			O2a	0.13205
**3**	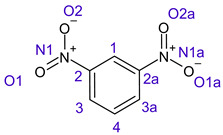	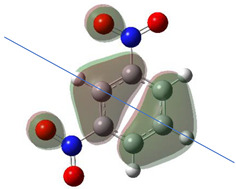	C1	0.1277
			C2	0.01980
			C2a	0.01976
			C3	0.04038
			C3a	0.04043
			C4	0.17606
			N1	**−0.01184**
			N1a	**−0.01183**
			O1	0.12144
			O1a	0.12141
			O2	0.12409
			O2a	0.12412
**4**	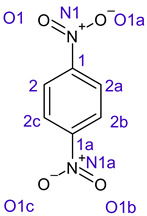	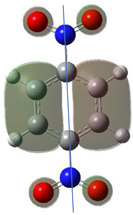	C1	−0.01979
			C1a	−0.01979
			C2	0.11729
			C2a	0.11730
			C2b	0.11729
			C2c	0.11730
			N1	−0.00706
			N1a	−0.00707
			O1	0.11958
			O1a	0.11958
			O1b	0.11957
			O1c	0.11957
**5**	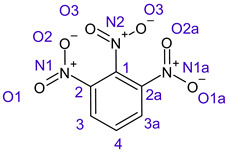	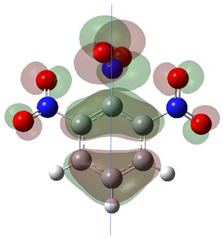 Imperfect m-symmetry	C1	0.05474
			C2	0.02281
			C2a	0.02283
			C3	0.02865
			C3a	0.02864
			C4	0.13450
			N1	**−0.00912**
			N1a	**−0.00911**
			N2	**−0.0108**
			O1	0.09177
			O1a	0.09179
			O2	0.07964
			O2a	0.07967
			O3	0.15307
			O3a	0.15315
**6**	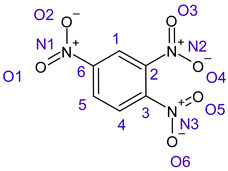	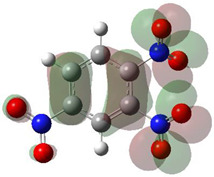 No symmetry	C1	0.00073
			C2	0.0556
			C3	0.03488
			C4	0.06947
			C5	0.07799
			C6	0.07799
			N1	**−0.01497**
			N2	**−0.00953**
			N3	**−0.00803**
			O1	0.09274
			O2	0.08489
			O3	0.13640
			O4	0.12613
			O5	0.13168
			O6	0.13468
**7**	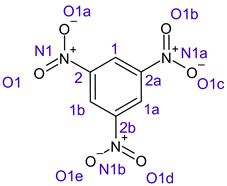	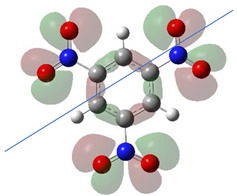 Broken C_3_-symmetry through the ring centre leaving just a perpendicular C_2_ axis	C1	0.00028
			C1a	0.00038
			C1b	0.00029
			C2	0.00021
			C2a	0.00017
			C2b	0.00021
			N1	**−0.00397**
			N1a	**−0.00079**
			N1b	**−0.00397**
			O1	0.19321
			O1a	0.10569
			O1b	0.10572
			O1c	0.19324
			O1d	0.14388
			O1e	0.14381
**8**	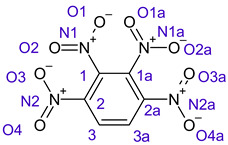	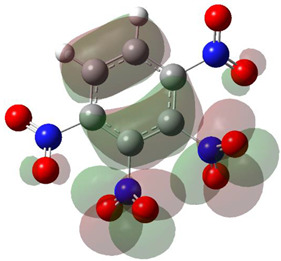 Imperfect mirror symmetry due to tilt in vicinal NO_2_-groups.	C1	0.00673
			C1a	0.05819
			C2	0.00021
			C2a	0.00011
			C3	0.03138
			C3a	0.05819
			N1	**−0.01071**
			N1a	**−0.01058**
			N2	**−0.00773**
			N2a	**−0.00830**
			O1	0.08932
			O1a	0.13085
			O2	0.08948
			O2a	0.12157
			O3	0.06601
			O3a	0.08383
			O4	**−0.01250**
			O4a	**−0.00949**
**9**	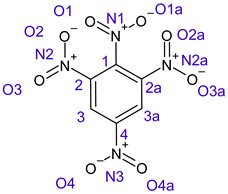	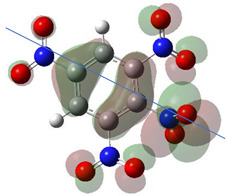	C1	0.02215
			C2	0.02222
			C2a	0.02215
			C3	0.01602
			C3a	0.01610
			C4	0.08079
			N1	**−0.00863**
			N2	**−0.00651**
			N2a	**−0.00651**
			N3	**−0.01386**
			O1	0.14493
			O1a	0.07638
			O2	0.07648
			O2a	0.1449
			O3	0.09631
			O3a	0.14493
			O4	0.08537
			O4a	0.08539
**10**	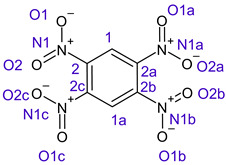	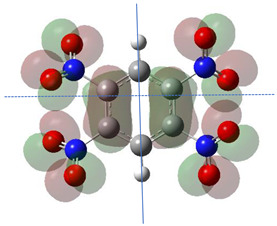	C1	**−0.00516**
			C1a	**−0.00866**
			C2	0.04254
			C2a	0.04265
			C2b	0.04246
			C2c	0.04257
			N1	**−0.00866**
			N1a	**−0.00866**
			N1b	**−0.00865**
			N1c	**−0.00865**
			O1	0.10561
			O1a	0.10587
			O1b	0.10564
			O1c	0.1059
			O2	0.0971
			O2a	0.09732
			O2b	0.09714
			O2c	0.0974
**11**	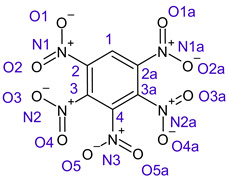	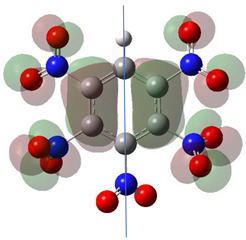	C1	**−0.00482**
			C2	0.06131
			C2a	0.06134
			C3	0.0212
			C3a	0.02118
			C4	**−0.00162**
			N1	**−0.01003**
			N1a	**−0.01003**
			N2	**−0.00739**
			N2a	**−0.00739**
			N3	**−0.01204**
			O1	0.09472
			O1a	0.09474
			O2	0.08082
			O2a	0.08084
			O3	0.10762
			O3a	0.11525
			O4	0.11527
			O4a	0.1076
			O5	**−0.00075**
			O5a	**−0.00074**
**12**	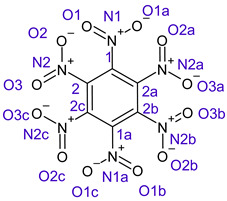	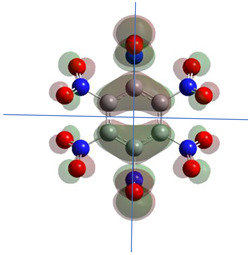 Even though at a first glance there seem to be 2 symmetry axes (shown as blue lines) data show that the system can better be described with a perpendicular C_2_-axis through the centre of the molecule.	C1	0.00763
			C1a	0.00763
			C2	0.05551
			C2a	0.00753
			C2b	0.05551
			C2c	0.00753
			N1	−0.00957
			N1a	−0.00955
			N2	−0.00955
			N2a	−0.00957
			N2b	−0.00895
			N2c	−0.00957
			O1	0.06550
			O1a	0.06399
			O1b	0.06417
			O1c	0.09901
			O2	0.0657
			O2a	0.0655
			O2b	0.06571
			O2c	0.0655
			O3	0.06417
			O3a	0.06399
			O3b	0.09903
			O3c	0.06399

## Data Availability

Data available upon request to the authors.

## Supplementary Materials

The supporting information can be downloaded at: https://www.mdpi.com/article/10.3390/ijms26010319/s1.
