# Supplementary material for: Systematic Approach to Negative Fukui Functions: Its Association with Nitro Groups in Aromatic Systems
Source: Int J Mol Sci. 2025 Jan 1;26(1):319. doi: 10.3390/ijms26010319 (PMC11719727; doi:10.3390/ijms26010319)
Supplement: Supplementary file 1 [file ijms-26-00319-s001.zip › ijms-3330899-supplementary.pdf]

**Dihedral angle**	**HOMO**	**Atom Number**	$f^{-}(\vec{r})$
0	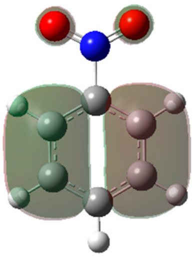	C1	-0.02617
		C2	0.16223
		C2a	0.17783
		C3	-0.02362
		C3a	0.17783
		C4	0.16222
		N	-0.01371
		O	0.09410
		Oa	0.09410
**Dihedral angle**	**HOMO**	**Atom Number**	$f^{-}(\vec{r})$
5	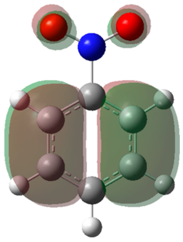	C1	-0.02841
		C2	0.16082
		C2a	0.17139
		C3	-0.02017
		C3a	0.18
		C4	0.15307
		N	-0.01425
		O	0.10843
		Oa	0.10917
**Dihedral angle**	**HOMO**	**Atom Number**	$f^{-}(\vec{r})$
10	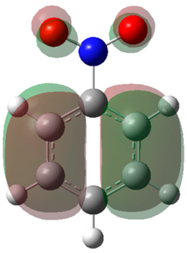	C1	-0.02823
		C2	0.16099
		C2a	0.17103
		C3	-0.01998
		C3a	0.18002
		C4	0.15282
		N	-0.01445
		O	0.10848
		Oa	0.10909
**Dihedral angle**	**HOMO**	**Atom Number**	$f^{-}(\vec{r})$
15	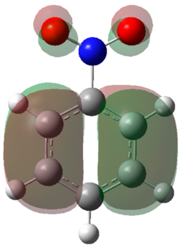	C1	-0.02808
		C2	0.16002
		C2a	0.1719
		C3	-0.01973
		C3a	0.17859
		C4	0.15383
		N	-0.01471
		O	0.10861
		Oa	0.10904
**Dihedral angle**	**HOMO**	**Atom Number**	$f^{-}(\vec{r})$
		C1	-0.0278

20	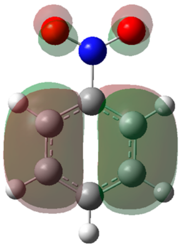	C2	0.15966
		C2a	0.17221
		C3	-0.01936
		C3a	0.17758
		C4	0.15459
		N	-0.01517
		O	0.10839
		Oa	0.10877
**Dihedral angle**	**HOMO**	**Atom Number**	$f^{-}(\vec{r})$
25	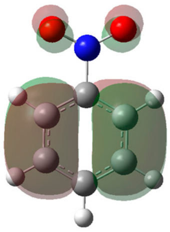	C1	-0.0275
		C2	0.1590
		C2a	0.1702
		C3	-0.0189
		C3a	0.1764
		C4	0.1535
		N	-0.0156
		O	0.1091
		Oa	0.1095
**Dihedral angle**	**HOMO**	**Atom Number**	$f^{-}(\vec{r})$
26	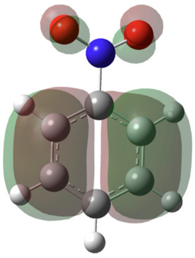	C1	0.006048
		C2	0.13341
		C2a	0.14569
		C3	0.02252
		C3a	0.15201
		C4	0.12881
		N	-0.01755
		O	0.12526
		Oa	0.11745
**Dihedral angle**	**HOMO**	**Atom Number**	$f^{-}(\vec{r})$
27	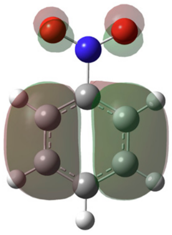	C1	0.0396
		C2	0.10782
		C2a	0.12117
		C3	0.06394
		C3a	0.12761
		C4	0.10412
		N	-0.01951
		O	0.14141
		Oa	0.1254
**Dihedral angle**	**HOMO**	**Atom Number**	$f^{-}(\vec{r})$
28		C1	0.07314
		C2	0.08223

	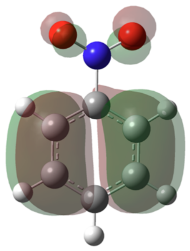	C2a	0.09666
		C3	0.10536
		C3a	0.10322
		C4	0.07944
		N	-0.02146
		O	0.15757
		Oa	0.13335
**Dihedral angle**	**HOMO**	**Atom Number**	$f^{-}(\vec{r})$
29	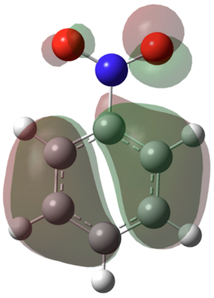	C1	0.10669
		C2	0.05664
		C2a	0.07214
		C3	0.14678
		C3a	0.07882
		C4	0.05475
		N	-0.02342
		O	0.17372
		Oa	0.1413
**Dihedral angle**	**HOMO**	**Atom Number**	$f^{-}(\vec{r})$
30	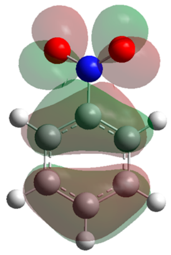	C1	0.14024
		C2	0.03105
		C2a	0.04763
		C3	0.1882
		C3a	0.05443
		C4	0.03006
		N	-0.02537
		O	0.18988
		Oa	0.14925
**Dihedral angle**	**HOMO**	**Atom Number**	$f^{-}(\vec{r})$
35	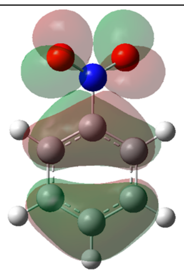	C1	0.14447
		C2	0.03154
		C2a	0.04873
		C3	0.19844
		C3a	0.05046
		C4	0.03014
		N	-0.0205
		O	0.16564
		Oa	0.17405
**Dihedral angle**	**HOMO**	**Atom Number**	$f^{-}(\vec{r})$
40		C1	0.13984
		C2	0.03034
		C2a	0.05085
		C3	0.19784

	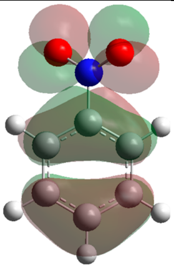	C3a	0.04623
		C4	0.03424
		N	-0.01874
		O	0.16883
		Oa	0.17284
**Dihedral angle**	**HOMO**	**Atom Number**	$f^{-}(\vec{r})$
45	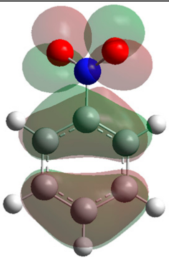	C1	0.13588
		C2	0.03149
		C2a	0.05038
		C3	0.19777
		C3a	0.0449
		C4	0.0361
		N	-0.01729
		O	0.16947
		Oa	0.17273
**Dihedral angle**	**HOMO**	**Atom Number**	$f^{-}(\vec{r})$
50	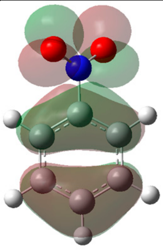	C1	0.13275
		C2	0.03226
		C2a	0.04994
		C3	0.19815
		C3a	0.04402
		C4	0.03776
		N	-0.01604
		O	0.1693
		Oa	0.1721
**Dihedral angle**	**HOMO**	**Atom Number**	$f^{-}(\vec{r})$
55	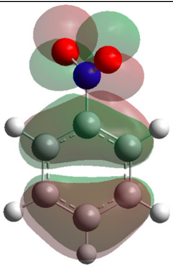	C1	0.13028
		C2	0.03379
		C2a	0.04932
		C3	0.19889
		C3a	0.04359
		C4	0.03858
		N	-0.01501
		O	0.16853
		Oa	0.17103
**Dihedral angle**	**HOMO**	**Atom Number**	$f^{-}(\vec{r})$
60		C1	0.1282
		C2	0.03533
		C2a	0.04873
		C3	0.19985

	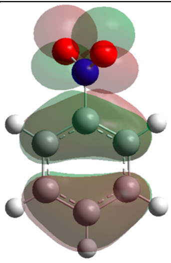	C3a	0.04317
		C4	0.03995
		N	-0.01421
		O	0.16742
		Oa	0.1696
**Dihedral angle**	**HOMO**	**Atom Number**	$f^{-}(\vec{r})$
65	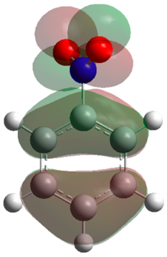	C1	0.12666
		C2	0.03682
		C2a	0.04817
		C3	0.20094
		C3a	0.04292
		C4	0.04118
		N	-0.0136
		O	0.1661
		Oa	0.16798
**Dihedral angle**	**HOMO**	**Atom Number**	$f^{-}(\vec{r})$
70	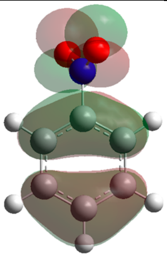	C1	0.12557
		C2	0.0382
		C2a	0.04765
		C3	0.20204
		C3a	0.0428
		C4	0.04221
		N	-0.01315
		O	0.16474
		Oa	0.16632
**Dihedral angle**	**HOMO**	**Atom Number**	$f^{-}(\vec{r})$
75	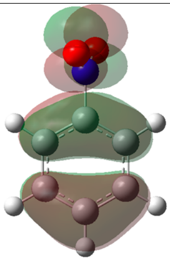	C1	0.12483
		C2	0.03944
		C2a	0.04715
		C3	0.20306
		C3a	0.0428
		C4	0.04301
		N	-0.01286
		O	0.16349
		Oa	0.16477
**Dihedral angle**	**HOMO**	**Atom Number**	$f^{-}(\vec{r})$
80		C1	0.12438
		C2	0.04049
		C2a	0.04667
		C3	0.20389

	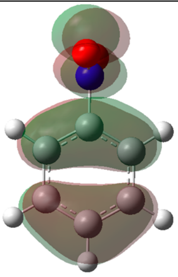	C3a	0.04292
		C4	0.04354
		N	-0.01268
		O	0.16249
		Oa	0.16347
**Dihedral angle**	**HOMO**	**Atom Number**	$f^{-}(\vec{r})$
85	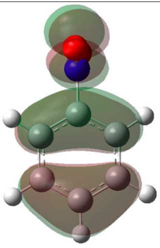	C1	0.12412
		C2	0.04131
		C2a	0.04621
		C3	0.20447
		C3a	0.04314
		C4	0.04377
		N	-0.01257
		O	0.16184
		Oa	0.16253
**Dihedral angle**	**HOMO**	**Atom Number**	$f^{-}(\vec{r})$
90	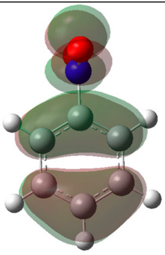	C1	0.12409
		C2	0.04169
		C2a	0.04601
		C3	0.20488
		C3a	0.04295
		C4	0.04418
		N	-0.01245
		O	0.16113
		Oa	0.16194

Figure S1 Table with correlation of dihedral angle and Fukui values on nitrobenzene

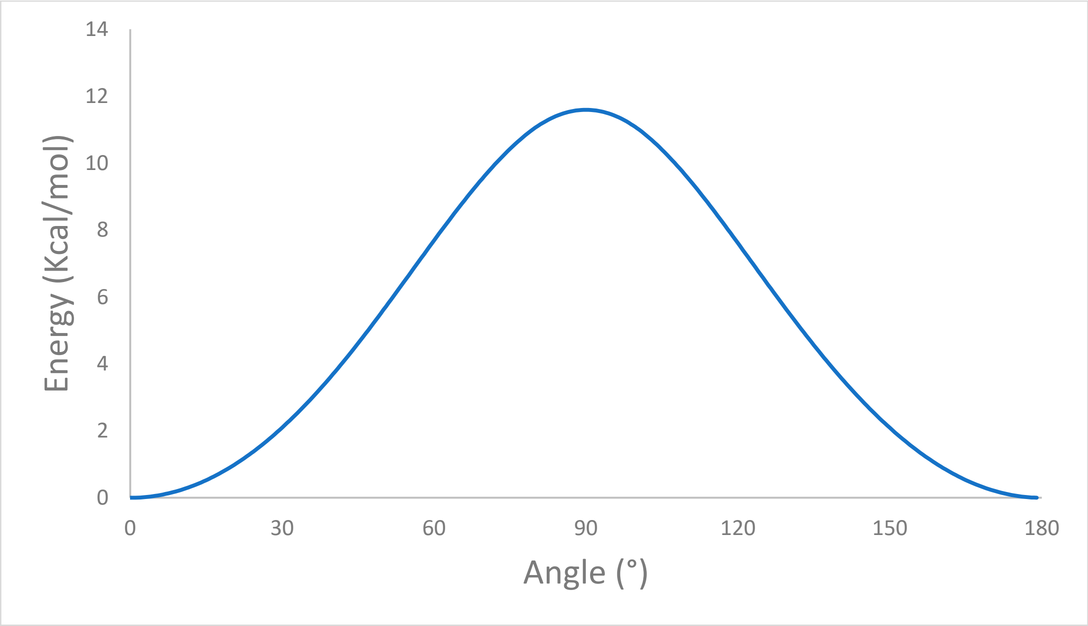

Figure S2. Relative energy vs dihedral angle in nitrobenzene

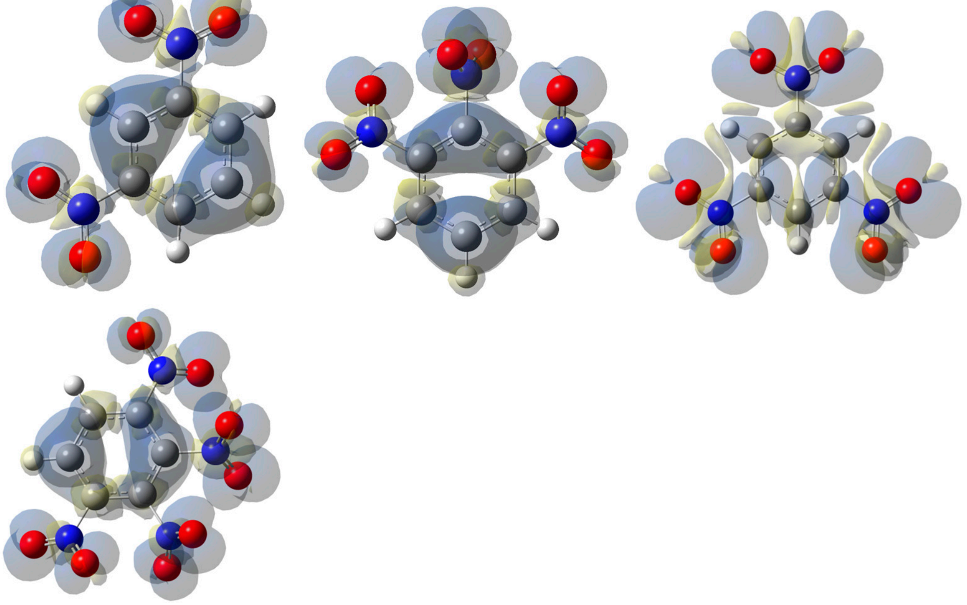

a) b) c) d)

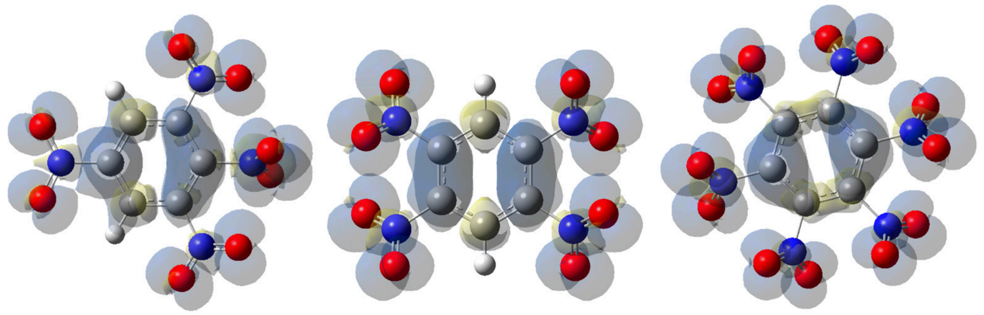

e) f) g)

Figure S3. Spin density: a) compound 3, b) compound 5 c) compound 7 d) compound 8 e) compound 9 f) compound 11 g) compound 12

Compound 8

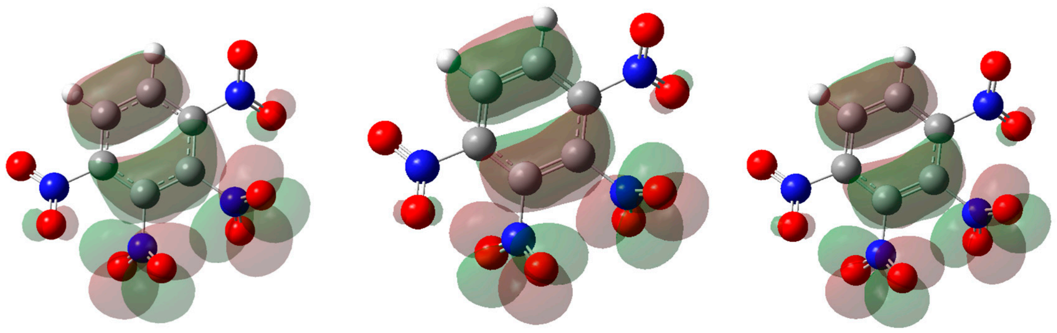

a b c

Figure S4. Comparison of the HOMO orbital (compound 8) with different functionals: a) B3LYP b) CAM-B3LYP c) ωB97X-D

	B3LYP	CAM-B3LYP	ωB97X-D
C1	0.00673	0.07113	0.007076
C1a	0.05819	0.05986	0.06017
C2	0.00021	0.00021	0.00041
C2a	0.00011	0.00011	0.00017
C3	0.03138	0.03138	0.05121
C3a	0.05819	0.05997	0.06119
N1	**-0.01071**	**-0.01147**	**-0.01104**
N1a	**-0.01058**	**-0.01213**	**-0.01218**
N2	**-0.00773**	**-0.00782**	**-0.00873**
N2a	**-0.0083**	**-0.0083**	**-0.00959**
O1	0.08932	0.08932	0.08953

O1a	0.13085	0.13378	0.14085
O2	0.08948	0.09048	0.07249
O2a	0.12157	0.11818	0.11076
O3	0.06601	0.04956	0.05011
O3a	0.08383	0.08383	0.08982
O4	**-0.0125**	**-0.0123**	**-0.0134**
O4a	**-0.00949**	**-0.00949**	**-0.0103**

Compound 11

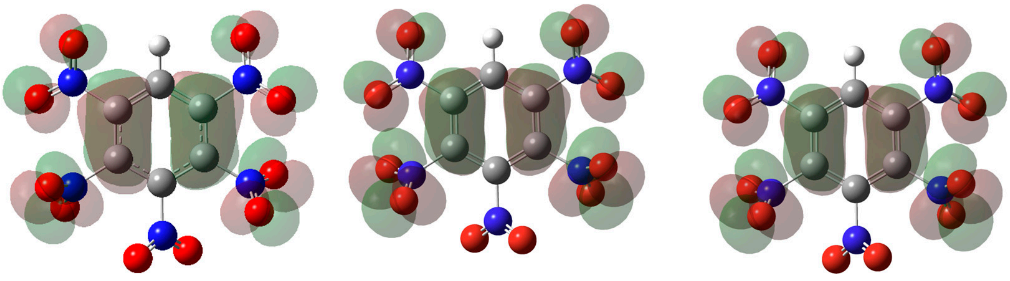

a b c

Figure S5. Comparison of the HOMO orbital (compound 11) with different functionals: a) B3LYP b) CAM-B3LYP c) ωB97X-D

	B3LYP	CAM-B3LYP	ωB97X-D
C1	**-0.00482**	**-0.00482**	**-0.00491**
C2	0.06131	0.06131	0.06121
C2a	0.06134	0.06134	0.06134
C3	0.0212	0.0212	0.0212
C3a	0.02118	0.02118	0.02118
C4	**-0.00162**	**-0.00162**	**-0.00162**
N1	**-0.01003**	**-0.01003**	**-0.01019**
N1a	**-0.01003**	**-0.01003**	**-0.01019**
N2	**-0.00739**	**-0.00739**	**-0.00739**
N2a	**-0.00739**	**-0.00739**	**-0.00739**
N3	**-0.01204**	**-0.01204**	**-0.01204**
O1	0.09472	0.09472	0.09472
O1a	0.09474	0.09474	0.09474
O2	0.08082	0.08082	0.08091
O2a	0.08084	0.08084	0.08098
O3	0.10762	0.1076	0.10811
O3a	0.11525	0.11525	0.11525
O4	0.11527	0.11527	0.11527
O4a	0.1076	0.1076	0.1076
O5	**-0.00075**	**-0.00075**	**-0.00075**

O5a	**-0.00074**	**-0.00077**	**-0.00081**

Compound	Atom	B3LYP angle (degrees)	ωB97X-D angle (degrees)
2	N1	38.94	40.21
	N2	41.17	42.15
6	N1	0.601	0.795
	N2	30.71	31.62
	N3	45.3	43.63
8	N1	31.72	33.6
	N2	60.47	59.87
	N3	60.89	60.34
	N4	33.26	35.02

Table S1. Fukui Function Values Calculated Using Different Functionals

Compound 2

	B3LYP	ωB97X-D
C1	0.06422	0.05722
C1a	0.06414	0.05914
C2	**-0.00185**	**-0.00195**
C2a	0.11475	0.13075
C3	0.11459	0.11459
C3a	**-0.00275**	**-0.00275**
N1	**-0.01226**	**-0.01226**
N1a	0.13211	0.11211
O1	**-0.01226**	**-0.01135**
O1a	0.13566	0.1122
O2	0.13575	0.11257
O2a	0.13205	0.10994

Table S2. Fukui Function Values Calculated Using Different Functionals

Compound 6

	B3LYP	ωB97X-D
C1	0.00073	0.00096
C2	0.0556	0.0673
C3	0.03488	0.03412

C4	0.06947	0.07677
C5	0.07799	0.07856
C6	0.07799	0.07999
N1	**-0.01497**	**-0.01518**
N2	**-0.00953**	**-0.01035**
N3	**-0.00803**	**-0.00898**
O1	0.09274	0.08831
O2	0.08489	0.07104
O3	0.13640	0.13147
O4	0.12613	0.12263
O5	0.13168	0.12338
O6	0.13468	0.12522

Table S3. Fukui Function Values Calculated Using Different Functionals
